# MetaCONNET: A metagenomic polishing tool for long-read assemblies

**DOI:** 10.1371/journal.pone.0313515

**Published:** 2024-12-03

**Authors:** Bingru Sun, Jian Guo, Hao Jin, Zijie Jin, Yaping Sun, Yuanchen Mao, Fuli Xie, Yun He, Zhihong Sun, Wei Li, Igor Ivanov, Hui Tian

**Affiliations:** 1 Axbio Biotechnology (Shenzhen) Co., Ltd., Shenzhen, China; 2 Key Laboratory of Dairy Biotechnology and Engineering, Ministry of Education, Inner Mongolia Agricultural University, Hohhot, China; 3 Peking University International Cancer Institute, Health Science Center, Peking University, Beijing, China; Brno University of Technology: Vysoke uceni technicke v Brne, CZECHIA

## Abstract

Accurate and high coverage genome assemblies are the basis for downstream analysis of metagenomic studies. Long-read sequencing technology is an ideal tool to facilitate the assemblies of metagenome, except for the drawback of usually producing reads with high sequencing error rate. Many polishing tools were developed to correct the sequencing error, but most are designed on the ground of one or two species. Considering the complexity and uneven depth of metagenomic study, we present a novel deep-learning polishing tool named MetaCONNET for polishing metagenomic assemblies. We evaluate MetaCONNET against Medaka, CONNET and NextPolish in accuracy, coverage, contiguity and resource consumption. Our results demonstrate that MetaCONNET provides a valuable polishing tool and can be applied to many metagenomic studies.

## 1. Introduction

Long-read sequencing technologies, led by Pacific Biosciences (PacBio) and Oxford Nanopore Technologies (ONT) platforms, can provide reads ranging from 1000 bp to over 100,000 bp, whereas the maximal read length for next-generation sequencing is around 300 bp [1–3]. With longer reads, assemblers can generate longer contigs across a variety of problematic regions like repetitive regions, which could be ambiguous referred by short read mapping tools. Thus, genome assembly using long reads can increase the continuity, reduce the assembly gaps, and fix the misassemblies such as translocations or inversions, making high-quality assembled genome more approachable. However, long-read sequencing shows systematic errors in between homopolymer sequences, thus introducing mismatches, insertions and deletions (InDels) to assemblies [4, 5], which may lead to frameshifts, cause the incorrect translation to protein sequences, and prevent the annotation and interpretation of assemblies [6].

Metagenomic assemblies polishing poses a significantly more complex challenge compared to refining single-species genomes. Metagenomic datasets inherently encompass a diverse collection of species with varying genome sizes, sequencing depths, and unique structural characteristics [7]. These factors make the genome polishing more intricate and computational resource demanding. To date, many polishing tools utilizing different algorithms have been developed for error correction. For example, Racon creates the consensus sequences with partial order graphs [8–10]. NextPolish uses Kmer Score Chain (KSC) and heuristic rules to find the consensus assembly, and short reads input is optional to improve the result [11]. Medaka (https://github.com/nanoporetech/medaka) and CONNET [12] are neural network-based tools to polish the assembly using only long reads [13, 14]. Despite the prevalence of well-established polishing tools designed for single-species genomes, the scarcity of polishing tools for metagenomics hinders the assembly and downstream analysis.

This neural network of CONNET contains two Bidirectional Recurrent Neural Network (BRNN) [15] models sequentially to rectify mismatches and InDels within assemblies. This model enables the capture of spatial relationship in sequences and yields superior accuracy and fewer deletions compared to other polishing tools. However, CONNET is not suitable for polishing of metagenomics for two reasons. First, CONNET’s models are trained on single-species genomes (*Escherichia coli* and Human chromosome 1). This limited training data spectrum might restrict its capability to capture the full range of variations encountered in metagenomes. Since BRNN relies on training data to learn sequence context, a broader range of species could significantly enrich the information captured by the model. Second, the current window size of 100bp in CONNET might limit the amount of contextual information considered during polishing. This limitation could potentially impact the effectiveness of CONNET on complex metagenomic assemblies.

To address these limitations and offer a comprehensive solution, we present MetaCONNET, a novel metagenomic polishing tool specifically designed to tackle the challenge of polishing the diverse and intricate metagenomic assemblies. MetaCONNET leverages the strengths of a well-established neural network method from CONNET and incorporates several enhancements. We conducted a rigorous evaluation of MetaCONNET, comparing its performance against other prominent neural network-based methods (Medaka, CONNET) and a state-of-the-art polishing tool, NextPolish. The results conclusively demonstrate that MetaCONNET delivers significant improvements in terms of accuracy and coverage for metagenomic assemblies.

## 2. Results and discussion

### 2.1 Overview of MetaCONNET

**Fig 1:**
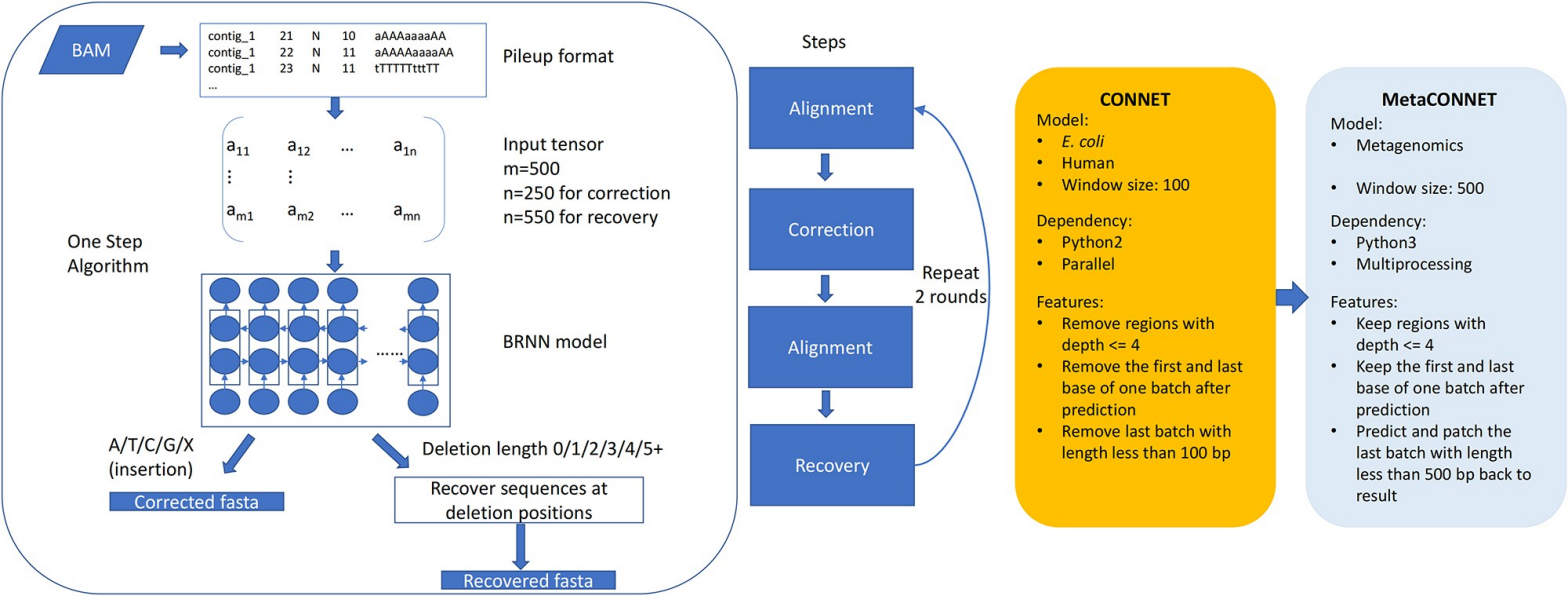
Overview of metagenomic polishing tool MetaCONNET. The figure presents the workflow of MetaCONNET and its enhancement features compared to CONNET.

MetaCONNET incorporates the following enhancements (Fig 1). First, to enrich the model’s ability of handling diverse metagenomic datasets, we curated a comprehensive training dataset encompassing four mock metagenomic community datasets (ONT R9 data), including HMP Microbial Mock Community B (HM-276D) [16], ZymoBIOMICS Mock Community Standards (Zymo10) [17], BMock12 Mock Community-12 isolates (Bmock12) [18], and ZymoBIOMICS gut microbiome standard (Zymo21) [14]; and one training dataset encompassing the ZymoBIOMICS High Molecular Weight (HMW) DNA Standard D6322 (Zymo mock) dataset (ONT R10.4 data) [19]. These datasets encompassing bacteria from a wide range of Phyla and Orders, reflect the inherent variability observed in real-world metagenomic samples and provide accurate reference sequences for training. Second, MetaCONNET employs a larger window size compared to CONNET, enabling it to capture a broader sequence context during polishing and enhance its effectiveness on complex metagenomic assemblies. Third, MetaCONNET is further optimized to handle low-coverage genomes, a frequent challenge in metagenomics, while maintaining efficient computational demands.

### 2.2 Metrics for metagenomic polishing evaluation

**Fig 2:**
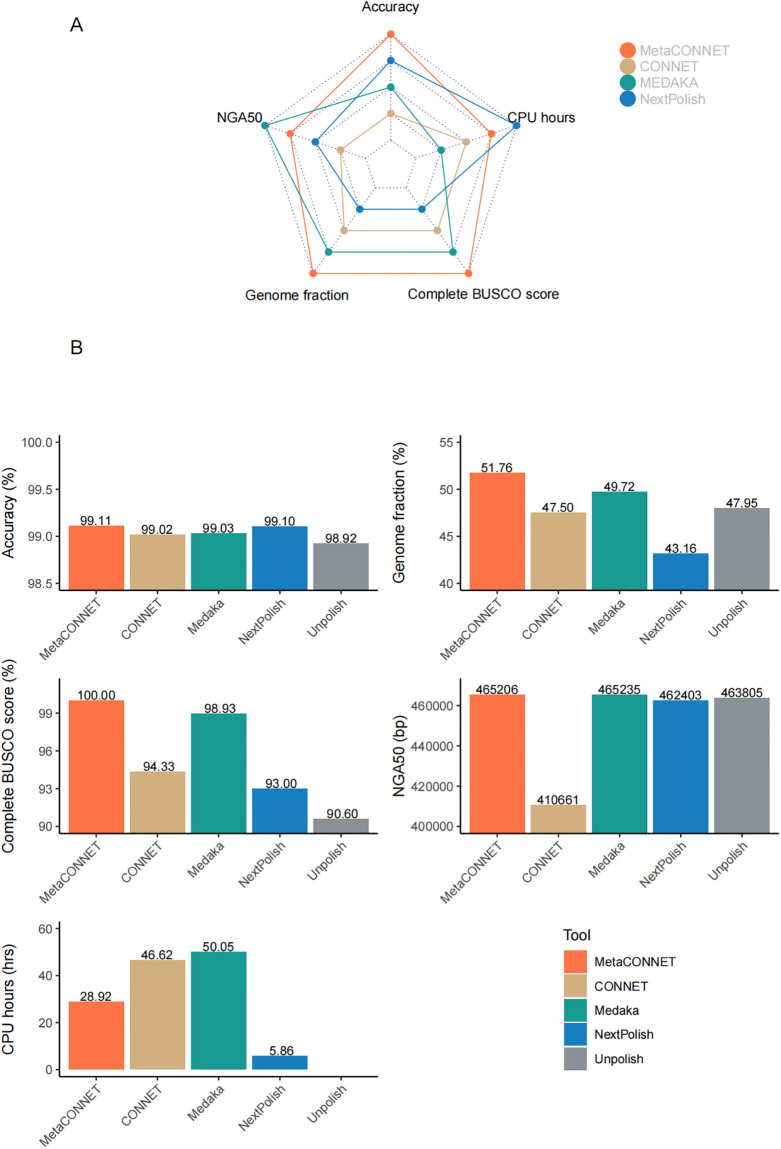
Performance of polishing tools (MetaCONNET, CONNET, Medaka and NextPolish) in five metrics. (A) The radar plot illustrates the comparative performance of four polishing tools (MetaCONNET, CONNET, Medaka and NextPolish) across five metrics on the total results of three datasets (MOCK1, MOCK2 and MOCK3). In the plot, higher values indicate superior rankings across metrics including Accuracy, Genome fraction, Complete BUSCO score [21] and NGA50. Conversely, lower CPU hour values signify higher rankings. MetaCONNET ranks top for Accuracy, Genome fraction and Complete BUSCO score, ranks the second for NGA50 and CPU hours. (B) Average performances of polishing tools across five metrics on three datasets.

Unlike most polishing tools tested on one or two representative species, we evaluated the performance of MetaCONNET against Medaka, CONNET and NextPolish, for the metaFlye assembly of three uneven microbial communities composed of up to 91 genomic microbial strains DNAs (MOCK1, MOCK2 and MOCK3) [20]. We comprehensively evaluated polishing tools’ performance on metagenomic data with five metrics, detailed list on Table 1 and shown the overall performance in Fig 2.

**Table 1:** Comparison of the polishing metrics of MetaCONNET, NextPolish, CONNET and Medaka.

Metrics	Accuracy	Genome fraction (%)	Complete BUSCO score (%)	Average NGA50 (bp)	CPU hours
Unpolish	0.9892	47.95%	90.60%	463805	/
MetaCONNET	**0.9911**	**51.76%**	**100.00%**	465206	28.92
NextPolish	0.9910	43.16%	93.00%	462403	**5.86**
CONNET	0.9902	47.50%	94.33%	410661	46.62
Medaka	0.9903	49.72%	98.93%	**465235**	50.05

Improving accuracy of assemblies is one of the main purposes of polishing tools. We first assessed the performance of accuracy, using ONT sequencing data, in three synthetic uneven DNA mocks (Table 2). Reducing the InDels error rate is crucial for nanopore sequencing polishing, due to homopolymer errors being the primary source of ONT sequencing errors [19]. MetaCONNET had consistently the best performance in InDels evaluation across the three datasets, with an average of 595.19 InDels per 100Kb (Fig 2 and Table 2). NextPolish outperformed in mismatches evaluation, with an average of 237.22 mismatches per 100Kb. The duplication ratio of NextPolish in MOCK3 is lower than in MOCK2 and MOCK1, which explains the further reduction in mismatches per 100Kb in MOCK3. However, for MetaCONNET, the duplication ratio remains relatively constant from MOCK1 to MOCK3, so its accuracy level stays more consistent compared to NextPolish (S1 Table). The overall ranking of accuracy is MetaCONNET (99.11%), NextPolish (99.10%), Medaka (99.03%) and CONNET (99.02%).

**Table 2:** Mismatches, InDels and accuracy comparison of polishing tools.

Dataset	Tool	Mismatches per 100 kbp	InDels per 100 kbp	Accuracy
MOCK1	Unpolish	353.37	809.98	0.9884
	MetaCONNET	320.73	**624.08**	**0.9906**
	NextPolish	**264.79**	680.69	0.9905
	CONNET	349.32	692.77	0.9896
	Medaka	328.09	723.74	0.9895
MOCK2	Unpolish	343.00	781.19	0.9888
	MetaCONNET	310.41	**593.09**	**0.9910**
	NextPolish	**264.75**	681.29	0.9905
	CONNET	343.38	675.33	0.9898
	Medaka	316.56	690.82	0.9899
MOCK3	Unpolish	236.35	692.20	0.9907
	MetaCONNET	241.28	**564.99**	0.9919
	NextPolish	**170.67**	602.21	**0.9923**
	CONNET	257.42	627.89	0.9911
	Medaka	222.31	617.28	0.9916
Total	Unpolish	315.00	764.69	0.9892
	MetaCONNET	293.19	**595.19**	**0.9911**
	NextPolish	**237.22**	657.92	0.9910
	CONNET	318.39	665.99	0.9902
	Medaka	292.57	680.25	0.9903

Genome fraction and Complete BUSCO score can be used to assessing the completeness of polishing result [21, 22]. Genome fraction is the total number of aligned bases in the reference divided by the genome size [23], which indicates the completeness in genome level. Complete BUSCO score is calculated as the proportion of complete genes in BUSCO markers, and higher Complete BUSCO score indicates higher quality and completeness in gene level. MetaCONNET has a clear advantage in these two metrics and is the only tool with a value of over 50% in genome fraction (51.76%). Genome fraction and Complete BUSCO score do not always agree, as can be seen from the unpolished data, where the genome fraction ranked in the middle, yet the complete BUSCO score is the lowest (Fig 2 and Table 1). MetaCONNET improved both coverage in genome level (Genome fraction) and in gene level (Complete BUSCO score) compared to unpolished data.

NGA50 is used to measure the contiguity of the assemblies, a reference-aware version of N50 metric [23]. A higher NGA50 value indicates a better contiguity and correctness of the assembly. MetaCONNET performs well on NGA50, which is much higher than CONNET (Fig 2 and Table 1). CPU hours is an important metrics of computational resource consumption. MetaCONNET has the best performance on CPU hours (28.92 h) among deep-learning based polishing tools (Fig 2). Considering metagenomics datasets are often of large amount of data, we optimized the computational performance through improved batch parallel design. For CONNET, contigs are split to batches with equal sequence sizes and these batches are polished in parallel using bash Parallel. During the model prediction step, polished sequences are still predicted sequentially instead of being processed in parallel. Therefore, we redesign the batch parallel of CONNET. Now all the sequences are first split into batches of size 100,000 bp and predicted in parallel using Python Multiprocessing package. The max process number is provided by the user. These enhancements guarantee optimal parallelization and utilization of resources during the polishing process, resulting in a further reduction in CPU hours.

Among all the tools, MetaCONNET has the lowest absolute number of introduced mismatches and indels across all datasets (S2 Table). MetaCONNET may be the most balanced choice among all tools, as it introduces fewer errors while improving accuracy in assemblies.

For R10.4 dataset, MetaCONNET leads in both the number of mismatches per 100 Kbp and genome fraction (S3 Table). Compared to MEDAKA, MetaCONNET surpasses it in both accuracy and genome fraction. Overall, these results are similar to those observed with the R9 datasets.

### 2.3 Species level comparison of accuracy and coverage among polishing tools

**Fig 3:**
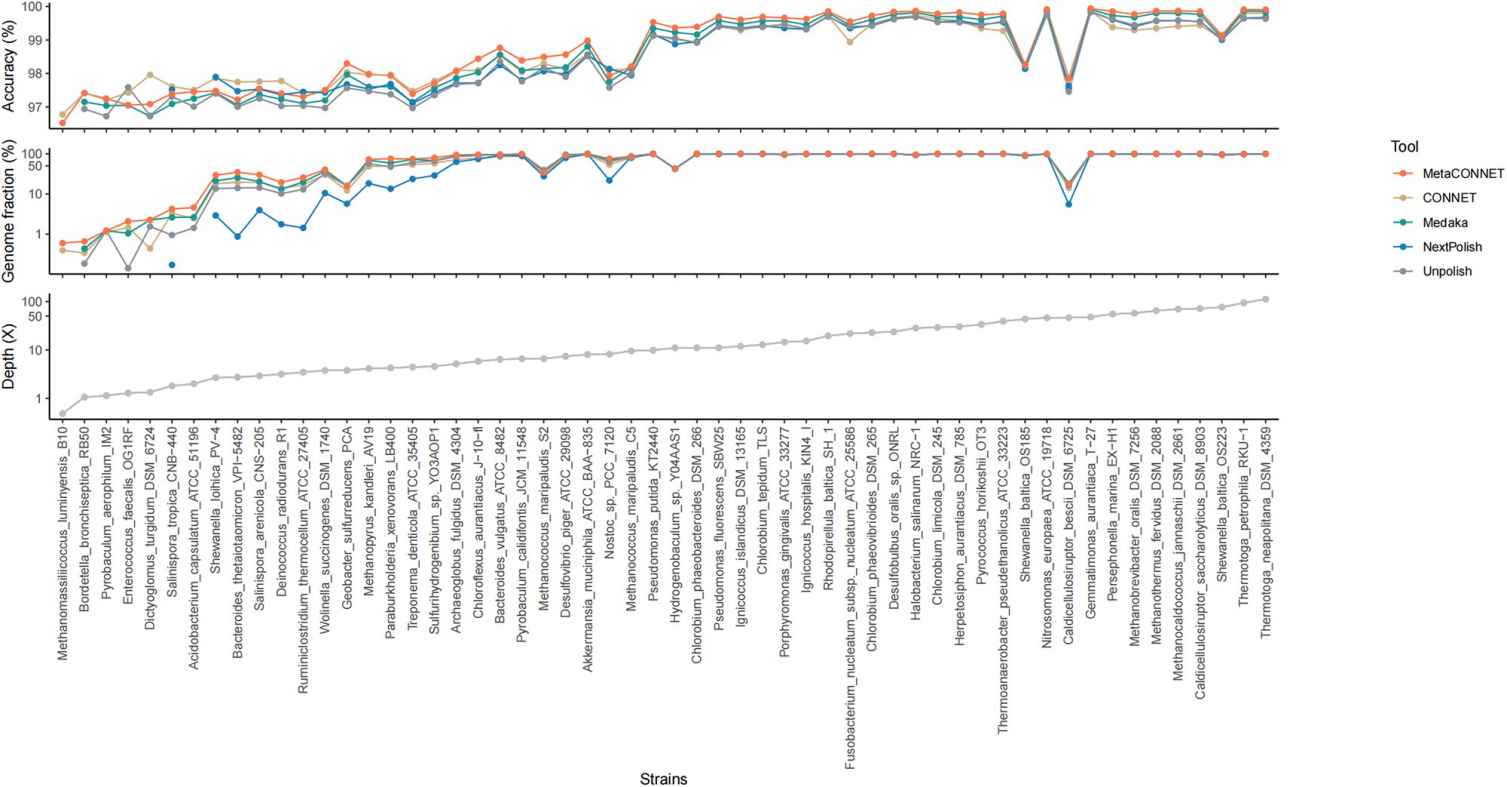
Microbial species (MOCK1) with genome fraction and accuracy. The line chart illustrates the values of Accuracy, Genome fraction, and Depth across species for MOCK1 assemblies polished by four tools (MetaCONNET, CONNET, Medaka and NextPolish), as well as the unpolished condition. Breaks in the lines indicating NA values. Species are arranged by depth from smallest to largest along the x-axis. Species with missing genome fraction in all of the conditions have been excluded, resulting in a total of 56 species represented in the chart.

The representative results of accuracy and genome fraction analyzed for each species (MOCK1) by four polishing tools are displayed in Fig 3. The advantages of MetaCONNET in terms of accuracy and genome fraction are demonstrated in most species in all three datasets (S4 Table). Generally, polishing tools can improve accuracy and quality of assembled results. However, for metagenomic sequencing data, the considerations are more complex. Microbial identification at species level remains a significant challenge in metagenomic study. Table 3 show the recovered species number and gene number (>95% ORFs) with different polishing tools. MetaCONNET and CONNET recovered most species number, total 170 in three testing set. NextPolish has the worst performance, recovered only 151 species, although it performs second best in the field of accuracy. In the part of recovered gene numbers, the advantage of MetaCONNET is even more significant. Gene number of MetaCONNET (127,856) is 1.18 times more than the second best (Medaka: 108,104), and 1.52 times more than the least (CONNET: 83,860). Regarding error levels, MetaCONNET demonstrates the lowest error rates in species identification and gene finding, as reflected by the Incorrect Species recovered and Incorrect ORFs ratios in MOCK1-3, compared to CONNET, Medaka, and NextPolish (S5 and S6 Tables).

**Table 3:** Recovered species number and gene number (>95% ORFs) among MetaCONNET, CONNET, Medaka and NextPolish.

Datasets	Class	MetaCONNET	CONNET	Medaka	NextPolish	Unpolish
MOCK1	Species	**56**	**56**	55	50	55
	>95% ORFs	**42,246**	27,809	36,346	36,543	36,954
MOCK2	Species	60	**61**	59	52	59
	>95% ORFs	**47,687**	31,149	38,674	32,030	32,231
MOCK3	Species	**54**	53	52	49	52
	>95% ORFs	**37,923**	24,902	33,084	25,640	25,521
Total	Species	**170**	**170**	166	151	166
	>95% ORFs	**127,856**	83,860	108,104	94,213	94,706

### 2.4 Analysis of species with different sequencing depths

**Fig 4:**
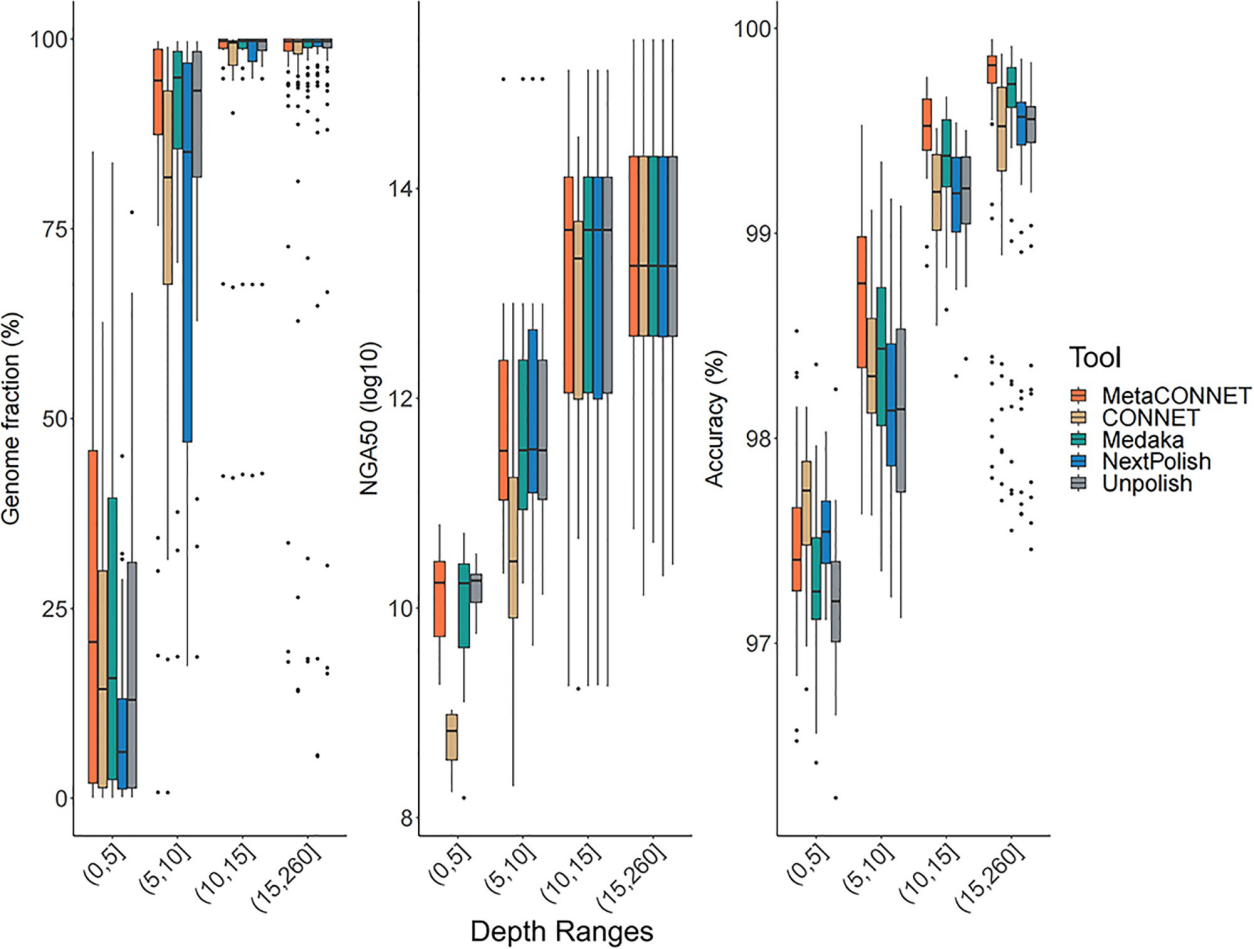
Genome fraction and accuracy according to sequencing depth by species. Boxplots summarize contig metrics for all available genomes across three mock datasets, separated into four depth range groups. Depth is derived from the alignment of raw reads to the reference genomes and represents the average depth for each genome.

One challenge in metagenomic study is to obtain relatively accurate and as complete as possible information for each species at different depths. We analyzed the performance of polishing tools at different depths in groups (Fig 4). It’s evident that across all four tools and in the unpolished condition, the average genome fraction increases with depth, as does accuracy. MetaCONNET consistently outperforms others in genome fraction across all depth ranges. When considering NGA metrics, MetaCONNET has comparable performance with the competitors, while CONNET consistently exhibits the lowest NGA values in the 0–5, 5–10, and 10–15 depth ranges. In terms of accuracy, MetaCONNET consistently performs best when the species’ depths > 5× but displays lower values in the 0–5 depth range. This can be attributed to the filters implemented within these two tools. CONNET employs a filter to eliminate low-coverage regions (below depth 4), while NextPolish has a similar filter at depth 3 at long read mode. These filters reduce the information load in low-depth species, leading to improved accuracy. Nonetheless, sequence information remains crucial for low-depth species, as a shortened genome length makes them challenging to specify. Hence, we opt not to implement any filter in our tool to maximize information retention for low-coverage species, albeit at the potential expense of accuracy in the 0–5 depth range. In conclusion, we achieve higher accuracy at equivalent depths and retain more information at lower depths, resulting in higher overall scores for both accuracy and genome fraction in metagenomic datasets.

### 2.5 Limitation and further improvement

Despite demonstrating significant promise in improving metagenomic assemblies’ accuracy and coverage, MetaCONNET presents opportunities for further optimization. A key challenge lies in its handling of insertion errors. During model training, insertions are inherently less frequent than other types of errors, leading to an imbalance in the model’s ability to correct them. Future iterations of MetaCONNET should prioritize enhancing its capacity to address insertion errors with greater accuracy.

MetaCONNET falls short of NextPolish in terms of reducing mismatches in assemblies. The pattern of mismatches errors is harder to be captured by the current model. In contrast, NextPolish utilizes a distinct mechanism that incorporates base quality information to address mismatch errors. To enhance MetaCONNET’s ability to classify mismatch errors effectively, it will be essential to integrate additional features into its model.

Furthermore, while MetaCONNET leverages a diverse range of bacterial species for training, the remarkable taxonomic diversity of bacteria in real-world scenarios surpasses the current scope of its training data. Consequently, generalizability to all prokaryotic species is not guaranteed, necessitating further comprehensive testing and validation across a wider spectrum of organisms.

Additionally, MetaCONNET is mainly trained based on ONT R9 sequencing data. Recently ONT released the R10 Kit, which improved sequencing accuracy with largely different error characteristics [24]. We have tested all the four tools on R10 data as is discussed previously (S3 Table). Since R10 is a new Kit with limited publicly available data, there is not sufficient diversity to fully test the performance of these models. Therefore, we would keep collecting more R10 data and tune MetaCONNET to further improve the model’s performance in the future.

Finally, while MetaCONNET currently outperforms other neural network-based models in terms of correction accuracy, a potentially synergistic approach could involve the integration of multiple polishing tools. Integrating multiple tools can provide a more comprehensive view of the data, ultimately enhancing the overall effectiveness of metagenomic analysis.

## 3. Conclusion

We introduced a novel polishing tool MetaCONNET, which is modified and retrained for metagenomic studies with long-read sequencing data. MetaCONNET has the best overall performance when evaluated across five metrics, especially on accuracy and genome fraction, which recovered the most species number in species level and most gene number in gene level. Meanwhile, MetaCONNET has good compatibility with other nanopore long-read sequencing platform and outperforms CONNET on Axbio sequencing data (S1 File,S1 Fig and S7 Table). By leveraging a tailored approach to training data, window size, and incorporating optimizations for low-coverage genomes and computational efficiency, MetaCONNET establishes itself as a powerful and versatile tool for researchers working with long-read metagenomic sequencing data.

## 4. Materials and methods

### 4.1 Dataset

Information on dataset is summarized in S8 Table. We collected four mock community datasets with ONT R9 sequencing data for model training and three mock community datasets for testing. The training mock datasets include HMP Microbial Mock Community B (HM-276D), ZymoBIOMICS Mock Community Standards (Zymo10), BMock12 Mock Community-12 isolates (Bmock12) and ZymoBIOMICS gut microbiome standard (Zymo21). Each training mock community dataset is comprised with a variety of bacteria species covering different Phylum and Order. We selected the mock datasets sourced from Meslier et al to validate and benchmark MetaCONNET. These mock datasets are synthetic uneven DNA mock samples and consist of three distinct samples MOCK1, MOCK2 and MOCK3, each with a different composition of strains (71 strains for MOCK1, 64 strains for MOCK2 and 87 strains for MOCK3), totaling 91 unique DNA strains mixed together [20]. For the R10.4 model, we used ZymoBIOMICS HMW (High Molecular Weight) DNA Standard D6322 (Zymo mock [19]) sequencing data for training, and created a synthetic mock dataset by combining the separate whole-genome sequencing data of four bacterial strains with uneven composition for testing (S9 Table) [25].

### 4.2 Genome sequencing and assembly

We downloaded all the four ONT sequencing datasets from NCBI SRA database: HM-276D (Accession: PRJNA630658 [16]), Zymo10 (Accession: PRJEB29504 [17]), Bmock12 (Accession: PRJNA496047 [18]) and Zymo21 (Accession: PRJNA804004 [14]). All the mock datasets are sequenced using ONT R9 flow cell with SQK-LSK109 kit, according to the metadata of the dataset from the Online Database. We use Porechop v0.2.4 (https://github.com/rrwick/Porechop) to remove adaptors to get clean sequencing data in FASTQ format. The FASTQ file of each mock dataset is assembled using Flye v2.9.1-b1780 using the parameter—meta—nano-raw -g 4m [26]. To test the robustness of the tool we didn’t filter the data with Q score threshold. The MOCK1-3 datasets were sequenced using MinION R9 flow cells with the SQK-LSK109 kit and the EXP-NBD103 barcoding kit, then basecalled and quality-trimmed with Guppy v2.3.1+9514fbc39 [20].

For R10.4 dataset, we downloaded the R10.4 PromethION sequencing FASTQ file from the SRA database (PRJEB48692) and downsampled it to a 25X depth relative to the reference length. The adaptors were removed using Porechop v0.2.4, and the 25X FASTQ data was assembled using Flye v. 2.9.1-b1780. We then mapped the 25X sequencing reads to the assembled contigs for use as the training sets and labeled the data according to the reference positions of the contigs. The training, validation, and testing datasets were divided in a 9:1:1 ratio. The R10.4 testing dataset was assembled using the same pipeline.

### 4.3 Model training

The input data for model training is made by aligning the sequencing reads to the draft assembly to obtain a bam which get converted to the input tensor. We label the input data using the alignment from draft assembly to the reference. All four mock datasets training data are combined and split to 10% as testing and 90% as training and validation. Training had run on a GPU for 200 epochs. We selected the models that has the best AUC performance on the training dataset and the testing dataset as the MetaCONNET metagenomics polishing model (S2 Fig).

### 4.4 Polishing method

The polishing method of CONNET is designed with two steps: Correction and Recovery. The correction step aims at fixing mismatches and insertions brought by sequencing or assembly errors. The recovery step aims at fixing the deletion gaps in the draft assembly. Each step composes of an alignment stage followed by one BRNN model prediction. We have not made major changes to the workflow of CONNET but focus on improving the model by increasing window size from 100 to 500 and using a larger dataset of metagenomic mock communities for training (Fig 1). However, during the development process, we found that CONNET may compromise the sequence information with rigorous filtering (depth > = 4) and incomplete batch (<100bp) lost at the end of contigs. To improve performance on metagenomics datasets, we removed the depth filter and re-predict the loss batch at the end of the contig and patch back to the sequence to keep the most sequence information (Fig 1).

### 4.5 Genome polishing and assessment

We use three polishing tools (CONNET v1.0, NextPolish v1.4.1, Medaka v1.7.3) to benchmark with our polishing model. All tools are run for two rounds of polishing and 12 core Intel(R) Xeon(R) Gold 5220R CPU @ 2.20GHz CPUs and 100G RAM. CONNET is run using model ecoli.R941 [12]. NextPolish [11] is set with task = best parameter. Medaka is run with model r941_min_high_g351. For polished genome evaluation, Quast v5.2.0 [27] is run with parameter—fragmented—min-alignment 500—min-identity 97—split-scaffolds—threads 12—min-contig 500 –circos. BUSCO v5.3.0 was run with bacteria_odb10 database [21, 28]. The gene prediction result of Prodigal [29] v2.6.3 is searched with the Uniprot 50 protein database using Diamond v2.1.8.162 [30]. The count of the protein with match length over 95% is recorded as >95% ORFs, which is an indicator of gene fragmentation. The software used for this project was summarized in the table (S10 Table).

## Supporting information

S1 Fig MetaCONNET metrics comparing with CONNET based on Axbio sequencing data. The radar plot illustrates the comparative performance of MetaCONNET, CONNET and Unpolish conditions across 4 metrics on the AxiLona AXP-100 assembly data tests. In the plot, higher values indicate higher rankings across all metrics. MetaCONNET demonstrates enhanced assembly quality across all four metrics compared to the original CONNET, showcasing its improved capability in rectifying errors in various Nanopore sequencing platforms.
(TIF)

S2 Fig ROC curves show the performance of MetaCONNET and CONNET. The ROC curve displays the true positive rate and false positive rate values at various threshold levels. The diagonal dashed line represents chance level or random guessing. For training, 10% of labeled data is reserved as the testing set. Both MetaCONNET and CONNET are tested using this dataset (MetaCONNET training, CONNET). Validation 1, 2, and 3 correspond to the labeled data from the MOCK1, MOCK2, and MOCK3 datasets, respectively. The results of MetaCONNET and CONNET validation are evaluated using these datasets (MetaCONNET Validation 1–3, CONNET Validation 1–3).
(TIF)

S1 Table Duplication ratio and total aligned length among Unpolish, MetaCONNET, NextPolish, CONNET, Medaka in MOCK1-3. (XLSX)

S2 Table Introduced errors ratio among MetaCONNET, NextPolish, CONNET, Medaka in MOCK1-3. (XLSX)

S3 Table Evaluation metrics of polishing assemblies using R10 models and R10 synthetic mock dataset. The top-performing tool for each metrics is indicated in bold and italic. The second-best tool is indicated in bold.
(XLSX)

S4 Table Species level performance on accuracy and genome fraction of four polishing tools. (XLSX)

S5 Table ORF analysis with MetaCONNET, CONNET, Medaka, NextPolish, Unpolish on MOCK1, MOCK2, MOCK3 datasets. (XLSX)

S6 Table Species analysis with MetaCONNET, CONNET, Medaka, NextPolish, Unpolish on MOCK1, MOCK2, MOCK3 datasets. (XLSX)

S7 Table Metagenomic assemblies polishing results comparison on Axbio sequencing data. (XLSX)

S8 Table Dataset summary. (XLSX)

S9 Table Information on R10 synthetic mock dataset. (XLSX)

S10 Table Description of the software and version. (XLSX)

S1 File Axbio platform library preparation and sequencing. (DOCX)
