# Supplementary figures and images for: MetaCONNET: A metagenomic polishing tool for long-read assemblies
Source: PLoS One. 2024 Dec 3;19(12):e0313515. doi: 10.1371/journal.pone.0313515 (PMC11614293; doi:10.1371/journal.pone.0313515)

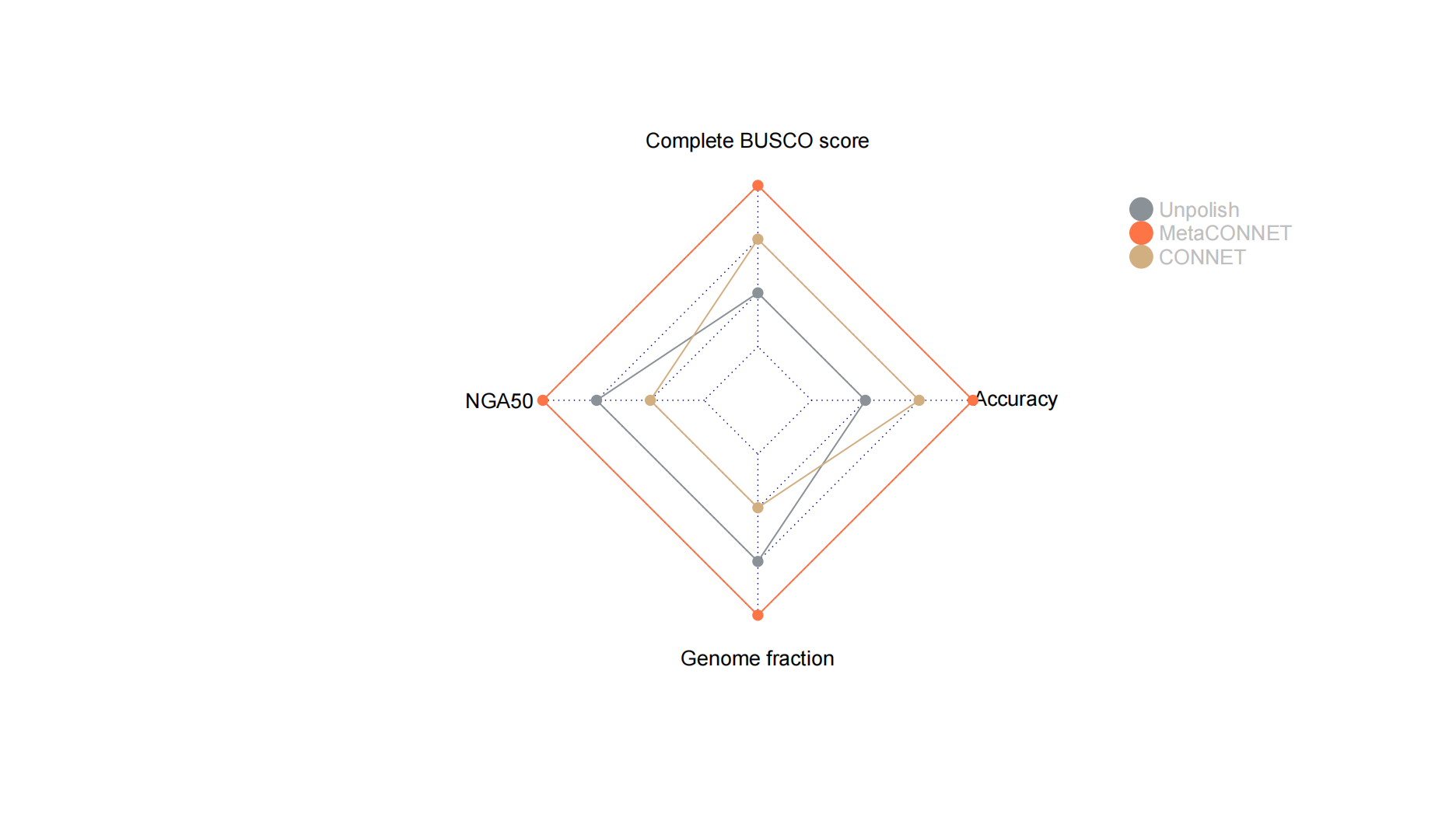

Supplement: S1 Fig — The radar plot illustrates the comparative performance of MetaCONNET, CONNET and Unpolish conditions across 4 metrics on the AxiLona AXP-100 assembly data tests. In the plot, higher values indicate higher rankings across all metrics. MetaCONNET demonstrates enhanced assembly quality across all four metrics compared to the original CONNET, showcasing its improved capability in rectifying errors in various Nanopore sequencing platforms. (TIF) [file pone.0313515.s001.tif]

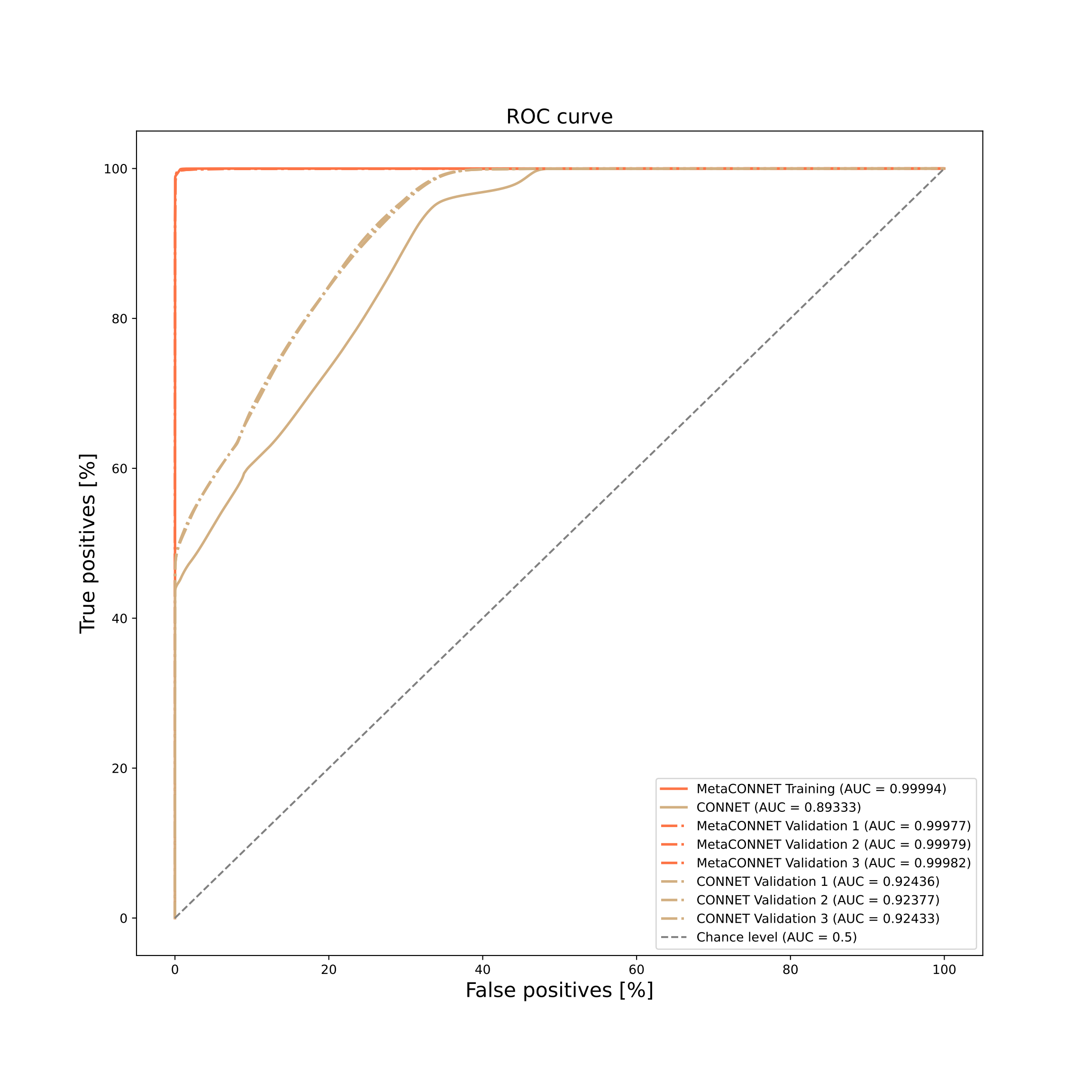

Supplement: S2 Fig — The ROC curve displays the true positive rate and false positive rate values at various threshold levels. The diagonal dashed line represents chance level or random guessing. For training, 10% of labeled data is reserved as the testing set. Both MetaCONNET and CONNET are tested using this dataset (MetaCONNET training, CONNET). Validation 1, 2, and 3 correspond to the labeled data from the MOCK1, MOCK2, and MOCK3 datasets, respectively. The results of MetaCONNET and CONNET validation are evaluated using these datasets (MetaCONNET Validation 1–3, CONNET Validation 1–3). (TIF) [file pone.0313515.s002.tif]
