# Supplementary material for: MetaCONNET: A metagenomic polishing tool for long-read assemblies
Source: PLoS One. 2024 Dec 3;19(12):e0313515. doi: 10.1371/journal.pone.0313515 (PMC11614293; doi:10.1371/journal.pone.0313515)
Supplement: S1 File — (DOCX) [file pone.0313515.s013.docx]

S1 File. Axbio platform library preparation and sequencing

The genomic DNAs of three strains of bacteria (*Bifidobacterium breve, Bifidobacterium animalis and Lactiplantibacillus plantarum*) were provided by Inner Mongolia Agricultural University.

600ng of gDNA from each sample was fragmented to construct the library according to the instructions of the AxiLona Prep K1.5 Library Preparation Kit (Axbio). Use the end repair module, ligation module, circularization module, and digestion module in the library prep set to perform DNA end repair, ligation, single stranded circularization, and digestion operations. Throughout the entire experiment, AMPure XP beads (Beckman Coulter) were used to purify and DNA was eluted using the recommended elution buffer in the instructions. Prepare the sequencing complex by referring to the detailed steps in the instruction manual of the AxiLona Seq 1.0 Sequencing Kit (Axbio). Load the sequencing complex and other reagents into the sample wells of the sequencing cartridge (Axbio) according to the instructions, and complete sequencing on the AxiLona AXP-100 sequencer.

The sequencer employs Nanopore Sequencing by Synthesis (NSBS) strategy, integrating innovative biochemistry with the mass production capabilities of semiconductor technology. The process utilizes advanced 12-inch copper dual damascene silicon and a microfluidic Bio-CMOS chip as its substrate. It detects signals through a unique AC impedance method with unbalanced ion composition *in trans* and *cis* sides, and used polymerase-nanopore biochemical system to achieve long-read sequencing. This design offers high sensitivity, rapid detection, and high throughput and with extremely low cost [1,2].

For this test, we have obtained an average of 46.6 Mb data for each strain of bacteria (*Bifidobacterium breve, Bifidobacterium animalis and Lactiplantibacillus plantarum*), the average sequencing depth for each strain of bacteria is 19.4X, and the average contig N50 is 149 Kb. The reads from three strains were processed to remove adaptors using Porechop v0.2.4. Following this, they were evenly combined to simulate metagenomic data, ensuring an equal total number of reads, with a minimum depth of 15X. The assembled fasta file was generated using CANU v2.2, with parameters set as follows: genomeSize=5m, minInputCoverage=1, minReadLength=100, minOverlapLength=10, and the -pacbio option.

**References**

1. Curtis-Joseph N, Peterson R, Brown [CE](https://loop.frontiersin.org/people/2740150), Beekman C, Belenky [P](https://loop.frontiersin.org/people/479193). Mouse diet and vendor impact microbiome perturbation and recovery from early-life pulses of amoxicillin. Front. Microbiomes. 2024;3: 1432202. doi:10.3389/frmbi.2024.1432202
2. Zhang, T, Li, H, Jiang, M, Hou, H, Gao, Y, Li, Y, Wang, F, Wang, J, Peng, K, Liu, Y.-X. Nanopore sequencing: flourishing in its teenage years, Journal of Genetics and Genomics. https://doi.org/10.1016/j.jgg.2024.09.007.
